# The Barretos short instrument for assessment of quality of life (BSIqol): development and preliminary validation in a cohort of cancer patients undergoing antineoplastic treatment

**DOI:** 10.1186/1477-7525-10-144

**Published:** 2012-11-29

**Authors:** Carlos Eduardo Paiva, Fernanda Capella Rugno, Bianca Sakamoto Ribeiro Paiva

**Affiliations:** 1 Departamento de Oncologia Clínica, Hospital de Câncer de Barretos, Rua Antenor Duarte Vilela, 1331, Bairro Dr Paulo Prata, CEP: 14784-400, Barretos, São Paulo, Brazil; 2 Barretos Cancer Hospital, Barretos, São Paulo, Brazil

**Keywords:** Quality of life, Questionnaire development, Reliability, Validity, Cancer

## Abstract

**Background:**

To be clinically useful, an instrument assessing health-related quality of life (HRQOL) should be easy to understand and quick to answer. Few instruments have been designed to be short, simple, and easily understandable by patients from all educational levels. The aim of the present study was to evaluate the psychometric properties of a brief general instrument developed to assess HRQOL.

**Methods:**

Results from a preliminary study regarding the initial development of the Barretos Short Instrument for Assessment of Quality of Life (BSIqol) with 80 cancer patients are presented. Out of all the patients, 59 completed the BSIqol on two occasions in order to evaluate the reproducibility test-retest. Validity analyses were done comparing scores from BSIqol with EORTC QLQ-C30 and Edmonton Symptom Assessment System (ESAS). In addition, BSIqol scores were analyzed in function of ECOG-PS, work activity, and financial income.

**Results:**

BSIqol demonstrated good internal consistency (Cronbach's α = 0.79) and adequate test-retest reliability, with intraclass coefficient correlation (ICC) varying from 0.736 to 0.946. There were adequate correlations between scores of BSIqol, EORTC QLQ-C30 and ESAS. The BSIqol was capable of discriminating between clinical subgroups, with different ECOG-PS and work activity. Patients completed the BSIqol in a median time <2 min. Only one patient reported some difficulty to answer the instrument.

**Conclusions:**

BSIqol seems to be a straightforward and useful instrument for rapidly assessing HRQOL from cancer patients. Further studies are necessary to evaluate BSIqol in different populations and also to assess its responsiveness and define its minimal clinically important differences.

## Background

Besides estimates of survival, it is necessary to include measures of health-related quality of life (HRQOL) to assess the outcomes of cancer treatment. Several tools have been developed for the assessment of HRQOL in oncology. In general, such tools are classified in generic, cancer-specific or cancer-site specific. The two most widely used questionnaires are the European Organization for the Research and Treatment of Cancer Quality of Life Questionnaire Core 30 (EORTC QLQ-C30) [1] and the Functional Assessment of Cancer Therapy-General (FACT-G) [2,3]. Both questionnaires are considered “core” instruments that can be supplemented by ‘specific modules’ targeting neoplasms, symptoms or specific treatments [3].

The main advantage of measuring HRQOL is the observation of the treatment clinical benefit according to patients’ own perspective. To be clinically useful, an instrument assessing HRQOL should be easy to understand and quick to be answered [4]. In this regard, currently, one can notice the lack of short instruments. The big challenge, as for creating instruments with small number of items, is the difficulty in turning them into multidimensional instruments. In addition to that, simplified instruments tend to have lower levels of reproducibility [5].

Another limiting factor for the routine use of HRQOL questionnaires is that patients with low educational level find them difficult to use. These patients have not been systematically included in the questionnaires validation studies. Few researchers [6,7] have dedicated time for the development of instruments targeted to this group of individuals, which, unfortunately, is still very common in Brazil.

The aim of the present study was to develop and validate a simplified instrument, comprised of only 6 items, which could be answered in a quick manner by people with different levels of education. Thus, the intention was to use general questions, with short explanatory texts and greater visual impact, using colors that would make it easier for the identification of values on a visual analogue scale ranging from 0 to 10.

## Methods

### Instrument development

The instrument was developed on the premise that the single item “feeling of well being”, from the ESAS [8] showed an adequate correlation with various domains of HRQOL [9]. Thus, the instrument has been designed in an attempt to use the understanding of the well-being concept from a patient’s standpoint. For the development of BSIqol, we considered HRQOL as the subjective perception, reported by the patient, about the impact of the disease and/or its treatment in their daily life, necessarily encompassing the dimensions of physical, psychological and social well-being [10].

Whereas HRQOL as a multidimensional construct, which necessarily includes the physical, emotional, social and functional aspects, we sought to create a simple instrument, with few items that would measure the HRQOL quickly and that could have applicability in daily oncology. The BSIqol is a generic instrument, created in a consensus meeting between two of the authors (CEP and BSRP), and improved along the subsequent steps.

Initially the BSIqol was answered by healthy people (n = 6), well-educated advanced cancer patients (n = 5), and than by oncology physicians (n = 5) and nursing professionals (n = 5). They could critique and suggest changes in the structure of the instrument in order to facilitate their understanding.

All the staff have formally rated the instrument using a standardized form. The BSIqol was initially designed in four ways: (1) using Likert type responses, (2) visual analogue scale, (3) visual numeric scale without colors, and (4) visual numeric scale with colors. Fourteen respondents (14/21; 67%) said they preferred the visual numeric scale, with added colors. No respondent reported having had difficulty understanding the instrument items. Among healthy people and cancer patients, there were no suggestions as to delete items, however, concerning the item 3, it was suggested to include the evaluations of optimism/pessimism. Two healthy people, a patient and a doctor, in order to make the instrument more clear, suggested removing words that made reference to some specific symptoms (fatigue, pain, vomiting, dyspnea) in the original instrument (item 2), and include more general items ("several unpleasant feelings"/"I don’t feel anything bad"). Among the five doctors, one suggested that the term "one week" could be changed from the beginning to the end of the sentences (items 1–3), and another doctor suggested changing the term "in general" for "as a whole" in item one. A nurse requested that items 5 and 6 were evaluated together. Another nurse suggested to be added to item 5, in parentheses, the information "bathing, dressing up, combing your hair, eating, etc.," not present in the original instrument. The BSIqol was reassessed once again by responders after the changes being carried out.

The instrument features six items arranged in figures, with questions written in large letters and short sentences. The BSIqol provides answers on a numerical scale ranging from 0 (worst) to 10 (best). We employed the color concept to facilitate the understanding of the respondents. The colors used were the colors often used in traffic lights (red, yellow and green). Answers may range from dark red (0), gradually moving towards yellow (medium) up to dark green (10) (Additional file 1 and Additional file 2).

Another feature of the instrument is that it contains several words and/or phrases designed to collect responses within the same domain. These words and/or phrases are located close to 0 and 10. For example, the item assessing the emotional domain (item 3), displays the words “sadness”, “anxiety”, “pessimism” and “unwilling to live” close to 0 (dark red). This same item, features the words “happiness”, “tranquility”, “optimism” and “great will to live” close to 10 (dark green). The words “terrible”, “more or less” and “excellent” were also included in this same item, respecting the positioning of the colors.

The items in the questionnaire being studied assess the HRQOL of patients during the period composed of the seven previous days. The original instrument is described in Additional file 1 and the version translated into English as Additional file 2. Questions for each item are listed below:

Item 1 - Think about your life as a whole. What grade would you give to your sense of well-being during the last week?

Item 2 – Think about your physical body. What grade would you give to your sense of physical well-being during the last week?

Item 3 - Think about your emotional side. What grade would you give to your sense of emotional well-being during the last week?

Item 4 - Think about the people you love, those that really matter to you. Overall, how is your relationship with them?

What grade would you give to your ability to:

Item 5 – Take care of yourself (bathing, dressing up, combing your hair, eating, etc.)

Item 6 – Work

With the intent of making it easier for comparisons between other instruments and to increase its clinical applicability, BSIqol scores were converted into a scale ranging from 0 to 100, with higher scores indicating better HRQOL, and calculated as follows:

$$\begin{array}{ccc} \text{BSIqol global} & = & \left[\left(\text{item}\;1+\text{item}\;2+\text{item}\;3+\text{Item}\;4\right)\right)\\ & & \left(\left(+\text{Item}\;5+\text{Item}\;6\right)/6\right]\times 100 \end{array}$$

BSIqol physical=item2×100

BSIqol emotional=item3×100

BSIqol social=item4×100

$$\text{BSIqol functional}=\left[\left(\text{item}\;5+\text{item}\;6\right)/2\right]\times 100$$

### Study setting and population

We interviewed 80 patients undergoing systemic antineoplastic treatment in the Department of Clinical Oncology at the Barretos Cancer Hospital (BCH) (Barretos, SP, Brazil), from April to June 2012. Patients were recruited in the chemotherapy/infusion room and the hormone therapy outpatient room. The inclusion criteria comprised of having been diagnosed with cancer and undergoing a systemic antineoplastic treatment, regardless of the histological type or stage of cancer. The exclusion criteria included the presence of significant neuropsychiatric disorder that could prevent patients from answering the study questionnaires, and also the refusal in taking part of the study. All patients agreed to participate in the study and signed a consent form. This project was approved by the Ethics Committee in Research at the BCH (HCB9489/2012).

### Data collection

#### Questionnaire administration

The questionnaires EORTC QLQ-C30, ESAS and BSIqol were applied by a single interviewer trained for the task. Regarding BSIqol, each item was printed on A4 paper (210 mm x 297 mm) and laminated afterwords. Items 5 and 6 were put together on a single sheet. The items were answered by the patients using special pens, which allowed for the reuse of the survey.

### Analytic strategies

#### Internal consistency and test-retest reliability

Reliability was assessed using Cronbach’s alpha coefficient, which should be ≥ 0.70 to be considered adequate [11].

A sample of 54 patients answered the BSIqol twice, to evaluate the test-retest reliability. To be submitted to retest, patients needed to be clinically stable and free of stressful events since the first evaluation. It was assessed using the intraclass correlation coefficient (ICC) with a retest interval of 2 h to 7 days of the initial assessment. The mean time (± SD) for the retest was 32.6 (± 65) hours. Test-retest reliability was considered adequate when ICC ≥ 0.70 [11].

#### Validity analysis

Construct validity was assessed using exploratory factor analysis of principal components with varimax orthogonal rotation. The Kaiser-Meyer-Olkin (KMO) Test and Bartlett’s Test of Sphericity were performed to indicate the degree of data susceptibility to factor analysis. To determine the number of factors to be extracted, we used the “scree test” proposed by Cattel and Jaspers [12].

Construct validity was assessed by measuring convergent and discriminative validity. For the convergent validation analysis, the BSIqol score was expected to be positively correlated with the global and functional scores of the EORTC QLQ-C30. Also, the BSIqol social was correlated with family income (measured in Brazilian minimum wages). Regarding discriminative validity, BSIqol was expected to be negatively correlated with the Total Symptom Distress Score (TSDS) of the ESAS (sum of the scores for pain, fatigue, nausea, anxiety, depression, drowsiness, anorexia and dyspnea), the ESAS emotional score (depression and anxiety mean scores) and the ESAS physical score (mean scores for pain, fatigue, nausea, drowsiness, anorexia, and dyspnea). In addition, BSIqol functional was also expected to be negatively correlated with Eastern Cooperative Oncology Group (ECOG) performance status scores.

Known-group validity was assessed by comparing the BSIqol scores according to different levels of ECOG-PS and also in relation to the working activity (active vs. inactive). Mann–Whitney and Kruskal-Wallis tests were used to examine group differences.

#### Feasibility

The time taken to answer the BSIqol was measured for 78 patients, who were asked about the likely issues they found when responding the instrument. The difficulties were categorized as (a) none, (b) some or (c) a lot. Another measurement examined was the missing data.

#### Sample size estimation

The sample size was estimated based on the assumption that would be adequate between three to twenty respondents for each single item in the instrument to be tested [13]; this equates to between 18 and 120 subjects for the BSIqol. In addition, 50 subjects may be considered an adequate sample size for test–retest reliability [14,15].

## Results

Eighty cancer patients were assessed and the most common primary tumors were breast (38/80; 47.5%) and prostate (20/80; 25%). The median age was 58 years (23–83 years). Most patients were women (57/80; 71%), Caucasian (65/80; 81%) and described inactivity at work (54/80; 68%). Regarding their marital status, 45 were married (45/80; 56%). The majority of patients were ECOG-PS 0 or 1 (69/80; 86%). TNM staging was IV in 38 patients (38/80; 48%). Half of the patients (40/80; 50%) were on chemotherapy and the other half was undergoing hormone therapy. In regard to their educational level, 50 patients (50/80; 62.5%) had low educational level, being regarded as illiterate and/or with only primary education completed (total ≤ 4 years of formal schooling). Patient characteristics are described in Additional file 3.

### Descriptive statistics

The median (25th-75th) scores of the BSIqol global were 70 (60.4-84.5), BSIqol physical 60 (50–80), BSIqol emotional 70 (40–87.5), BSIqol functional 75 (60–90) and BSIqol social 90 (80–100).

### Internal consistency and test-retest reliability

The Cronbach’s alpha of the instrument was 0.79 (Table 1) and ICC varied from 0.736 to 0.946 (Table 2).

**Table 1:** Internal consistency of BSIqol (N = 80)

**BSIqol**	**Mean (SD)**	**Median (25**^**th**^**-75**^**th**^**)**	**Cronbach’s alpha if item deleted**	**Cronbach’s alpha**
*Global*	70.2 (16.5)	70 (60.5–84.5)		0.790
*Items*				
1	6.6 (2.16)	6.5 (5–8)	0.737	
2	6.2 (2.30)	6 (5–8)	0.746	
3	6.05 (2.72)	7 (4–8.5)	0.726	
4	8.62 (2.01)	9 (8–10)	0.808	
5	8.98 (1.91)	10 (9–10)	0.780	
6	5.66 (2.83)	6 (3.5-8)	0.753	

**Table 2:** Test-retest reliability analysis of BSIqol (N = 54)

**BSIqol**	**ICC**	**95% CI**	**p-value**
Global	0.946	0.907–0.969	<0.001
Physical	0.900	0.828–0.942	<0.001
Emotional	0.851	0.744–0.913	<0.001
Functional	0.932	0.883–0.960	<0.001
Social	0.736	0.553–0.847	<0.001

Legend: *ICC* intraclass correlation coefficient.

### Validity analysis

The KMO index (0.80) indicated that there was sufficient level of factorability. The Bartlett's sphericity test (p < 0.001) indicated that the correlation matrices were not identical to the factor structure matrices. Both tests revealed that data were appropriate for factor analysis. The “scree test” identified three factors that explained a total variance of 78.5%. Factor 1 was considered as “well-being”, factor 2 as the “functional” and factor 3 as “social”. However, for the analysis of the validation criteria of the BSIqol described next, and because it is more useful clinically, we chose to continue considering the Factor 1 divided as BSIqol emotional and BSIqol physical (Table 3).

**Table 3:** Principal component analysis of BSIqol items

**BSIqol**	**Factor 1**	**Factor 2**	**Factor 3**
**Item 1**	0.828		
**Item 2**	0.871		
**Item 3**	0.725		
**Item 4**			0.966
**Item 5**		0.930	
**Item 6**		0.621	
**% of variance**	49.9	15.5	13.1
**Cronbach’s *α***	0.80	0.60	NA

*p < 0.001. Legend: *NA* not applicable. Factorial loads <0.4 were omitted.

There was a high correlation between the BSIqol global score and global health score of the EORTC QLQ-C30 (r = 0.767; p < 0.001). In the same manner, the BSIqol global correlated itself well with the global functional scale (r = 0.809; p < 0.001) and global symptoms (r = −0.678; p < 0.001) (Table 4).

**Table 4:** Spearman correlation analysis between BSIqol domains and EORTC QLQ-C30

**EORTC QLQ-C30**	**BSIqol**				
	**Global**	**Physical**	**Emotional**	**Functional**	**Social**
*Global health status*	0.767**	0.639**	0.620**	0.534**	0.472**
*Function global*	0.809**	0.724**	0.725**	0.553**	0.354**
Physical functioning	0.664**	0.669**	0.465**	0.579**	0.205
Role functioning	0.704**	0.715**	0.560**	0.529**	0.272*
Emotional functioning	0.658**	0.440**	0.774**	0.317**	0.422**
Cognitive functioning	0.288	0.335**	0.363**	0.058	0.168
Social functioning	0.364**	0.330**	0.291**	0.296**	0.223*
*Symptom global*	−0.678**	−0.701**	−0.565**	−0.477**	−0.259*
Financial difficulties	−0.328**	−0.205	−0.228*	−0.304**	−0.323**

*p < 0.05 **p < 0.01. **_**Underline indicates a pair of scales that should correlate theoretically.

The BSIqol physical was the domain that best correlated with the physical functioning subscales (r = 0.669; p < 0.001) and role functioning (r = 0.715; p < 0.001) (Table 4).

BSIqol emotional was the domain with the highest value for “r” in relation to emotional functioning subscale of the EORTC QLQ-C30 (r = 0.774; p < 0.001) (Table 4).

BSIqol functional provided adequate correlation with the global functional domain (r = 0.553; p < 0.001), physical functioning (r = 0.579; p < 0.001) and role functioning (r = 0.529; p < 0.001) (Table 4).

While having a low correlation coefficient, the BSIqol social correlated significantly with the social functioning subscale (r = 0.223; p < 0.05) and with the financial difficulties domain (r = −0.323; p < 0.001) (Table 4).

Regarding the ESAS, all domains of BSIqol correlated negatively with TSDS. However, the highest correlation coefficients were related to the BSIqol global (r = −0.708; p < 0.001), BSIqol physical (r = −0.679; p < 0.001) and BSIqol emotional (r = −0.706; p < 0.001). As expected, the BSIqol domains that best correlated with the ESAS physical were BSIqol global (r = −0.670; p < 0.001) and BSIqol physical (r = −0.699; p < 0.001). Similarly, the field that best correlated as ESAS emotional was BSIqol emotional (r = −0.593; p < 0.001) (Table 5).

**Table 5:** Spearman correlation analysis between BSIqol domains and ESAS

**ESAS**	**BSIqol**				
	**Global**	**Physical**	**Emotional**	**Functional**	**Social**
TSDS	−0.708**	−0.679**	−0.706**	−0.384**	−0.343**
Physical-ESAS	−0.670**	−0.699**	−0.588**	−0.421**	−0.261*
Emotional-ESAS	−0.482**	−0.371**	−0.593**	−0.249**	−0.374**

*p < 0.05 **p < 0.01. _Underline indicates a pair of scales that should correlate theoretically.

Legend: *ESAS* Edmonton Symptom Assesment System.

BSIqol scores were negatively correlated with the ECOG-PS. As expected, the domain with the highest correlation coefficient was BSIqol functional (r = −0.714; p < 0.001) (Additional file 4).

In an interesting way, BSIqol social was the only one that correlated in a statistically significant way with patients’ family income (r = 0.208; p < 0.05) (Additional file 4).

All domains of BSIqol, with the exception of BSIqol social, showed different medians between the different statuses of ECOG-PS. There was a progressive worsening of the BSIqol scores such as global, physical, emotional and functional as the functional performance worsened (Table 6).

**Table 6:** Median scores (25th-75th) of BSIqol domains between different Performance Status according to ECOG

**BSIqol**	**ECOG - PS**			
	**0 (n = 34)**	**1 (n = 35)**	**2/3 (n = 11)**	**p-value**
**Global**	85 (72.5–91.7)	65 (57.5–73.3)	53.3 (38.3–67.5)	**<0.0001**
**Physical**	80 (60–90)	50 (50–70)	40 (25–60)	**<0.0001**
**Emotional**	75 (60–90)	50 (40–80)	30 (10–65)	**<0.0001**
**Functional**	92.5 (80–100)	70 (60–75)	55 (42.4–75)	**<0.0001**
**Social**	100 (90–100)	90 (80–100)	80 (50–100)	0.108

Legend: *ECOG* –*PS* Eastern Cooperative Oncology Group performance status.

Regarding the working activity, one could have observed that patients in active work had higher global, emotional and functional BSIqol scores. We emphasize the difference found in the median scores of BSIqol functional among individuals in active and inactive work (90 and 70, respectively) (p < 0.001) (Table 7).

**Table 7:** Median scores (25th-75th) of BSIqol domains between work active and inactive patients

**BSIqol**	**Work activity**		
	**Active**	**Inactive**	**p-value**
**Global**	78.3 (65.8–89.1)	68.3 (54.2–76.7)	**0.005**
**Physical**	70 (45–85)	60 (50–80)	0.554
**Emotional**	70 (55–90)	60 (30–80)	**0.033**
**Functional**	90 (75–97.5)	70 (55–80)	**<0.001**
**Social**	100 (90–100)	90 (70–100)	0.198

*p < 0.05 **p < 0.01.

### Feasibility

The median time to answer BSIqol was 1:47 min (0:48–4:53). Out of the 78 patients interviewed, only one (1/78; 1.3%) reported having some difficulty in answering the instrument. The same patient, who was illiterate, reported having great difficulty in answering the EORTC QLQ-C30, and mentioned that it was easier to answer the BSIqol in relation to the EORTC QLQ-C30, due to the fact that the survey instrument displayed colors.

Regarding the EORTC QLQ-C30, 4 patients reported having some difficulty and 1 patient had great difficulty answering it (completely with difficulty 5/78; 6.4%). Out of these 5 patients, 2 were illiterate and 3 of them had had ≤ 4 years of formal schooling.

There was no missing data (“missing values”) in relation to BSIqol.

## Discussion

The authors developed a new instrument to measure HRQOL. The BSIqol was designed to be a generic questionnaire and was validated in a sample of cancer patients. The great advantage of the proposed instrument is its likely clinical usefulness since it was answered quickly and without difficulty by the interviewees, even in a population having low educational level where approximately 60% had less than four years of formal schooling and 10% were illiterate.

The BCH is exclusively dedicated to oncology and serves a needy population in terms of financial resources, originated from all corners of Brazil. The development of BSIqol comes to fill a gap in clinical practice observed by the authors of this research, where many patients have difficulty answering instruments that assess “patient-reported outcomes”, even instruments that have been appropriately validated in the country. This is probably because the population of low socioeconomic level treated in the hospital does not reflect the populations subjected to validation studies often conducted around the country.

Considering HRQOL within a multidimensional concept, the instruments proposed for its measurement must assess at least the physical, emotional, functional and social aspects [16]. To this end, the tools developed are mostly long and sometimes complex, or too stressful for some individuals.

The main advantage in measuring HRQOL is the observation of clinical benefits on the treatment from the patient’s own perspective. In oncology, the two most widely used instruments in research are EORTC QLQ-C30 and FACT-G [3]. The EORTC QLQ-C30, comprised of 30 items, and FACT-G, with 28 items, are usually answered within 5 to 10 min. The BSIqol was answered in a median time of <2 min, which was very relevant, considering the low socioeconomic level of the population assessed in this study.

The search for instruments that would measure the HRQOL with few items is not new [17-19]. Some instruments with few items proved to be valid in some specific situations; however, they are still rarely used [20-22]. Data in the literature suggest that short questionnaires have higher response rate (when mailed to patients) and lower rates of unanswered items, compared with long questionnaires [23,24]. Moreover, short questionnaires would possibly be less of a burden to patients and would facilitate the operational logistics involved in health services [25].

The reduction in items from longer questionnaires in order to make them shorter has proved to be feasible in previous studies, and the short instruments did not lose their psychometric characteristics [26,27]. One instrument worth mentioning is the Quick-FLIC, with 11 items, whose total score showed good correlation with the EORTC QLQ-C30 global health, with correlation coefficients ranging from 0.71 to 0.77 [28]. The global score of the Quick-FLIC may vary from 0 to 100 and it was more clinically relevant than the scores of individual items. Similarly, the BSIqol global score correlated with the EORTC QLQ-C30 global health with a high correlation coefficient (r = 0.76). As with the Quick-FLIC global score, we believe the global score is the most useful index extracted from BSIqol for future studies and for use in clinical practice.

Some studies suggest that social support may interfere with HRQOL [29,30]. The BSIqol social domain specifically assessed interpersonal relationships based on the theoretical premise that a good relationship with people considered important to patients would be associated with good social support. The BSIqol social correlated significantly with the social functional domain of the EORTC QLQ-C30, however, the correlation coefficient was found to be low (r = 0.223). This can be explained in that the EORTC QLQ-C30 assesses the social impact on HRQOL caused by cancer or its treatment; the BSIqol, however, upon assessing the interpersonal relationships, does not associate them with the disease or its treatment. The authors believe that problems in interpersonal relationships may impact HRQOL regardless whatever its cause may be. Interestingly enough, we observed that the BSIqol social scale correlated with the financial difficulties subscale and also correlated negatively with the family income of patients. This finding points to the association between problems in interpersonal relationships and financial difficulties. Financial difficulties secondary to cancer are common [31] and may interfere with HRQOL [32].

The BSIqol emotional showed high correlation coefficient with EORTC QLQ-C30 emotional functioning domain (r = 0.775) and also with the ESAS-emotional (r = −0.593). Similarly, the BSIqol physical scale correlated with the physical functioning (r = 0.669), role functioning (r = 0.715), global symptoms (r = −0.701) and the ESAS-physical (r = −0.699). These correlations point to an adequate validation of the physical and emotional BSIqol domains.

In spite of the adequate correlation coefficients (r > 0.5) displayed by BSIqol functional when correlated with the functional domain of EORTC QLQ-C30, the BSIqol global showed even higher rates. However, in order to validate the BSIqol functional, we found the highest correlation with ECOG-PS when we correlated the functional performance with BSIqol functional (r = −0.714). Furthermore, functional BSIqol scores were significantly higher in patients who maintained work activity, in relation to those inactive at work. In this analysis, the BSIqol functional was the domain most associated with work activity.

In addition to the color concept, the BSIqol uses short and simple sentences, with large letters. Each item is shown on a different page, except for items 5 and 6 (functional domain), shown on a single page because they are interrelated with one another. The authors believe that such “layout” facilitates the understanding of patients, since only one of the respondents reported some difficulty to understand it. The Moorehead-Ardelt Quality of Life Questionnaire is an instrument originally developed to measure HRQOL in obese patients after surgery. This questionnaire uses symbols and colors that facilitate the understanding of patients. It is believed that such peculiarities made the instrument have a wide spread use in certain corners of the world due to its convenience and ease of being understood [33]. In the present study, we chose the colors red, yellow and green, because they are related to traffic signs, possibly being considered as a universal language.

This preliminary study has some limitations. One of them is that the questionnaire responsiveness of was not evaluated. Another limitation is the lack of data regarding the minimum difference in scores that are clinically significant to the patient. While this study is preliminary in nature, further studies are necessary to clarify the mentioned limitations.

We believe that the major contribution posed by this study is to show the feasibility as for the development of very short instruments with good reliability. Furthermore, the characteristics of the instrument (use of colors, one item per page, several words/phrases showing the same domain, etc.) could be employed as a model for future instruments.

## Conclusions

We developed a simplified HRQOL measuring tool containing only six items, which is easily understood by patients and easy to be applied. The instrument has demonstrated adequate psychometric characteristics, with good internal consistency and criterion validation. Given the aforementioned characteristics, the BSIqol displays relevant potential for application in oncology practice. Further studies are needed to evaluate the psychometric characteristics in other populations and assess the instrument responsiveness.

## Abbreviations

HRQOL: Hhealth-related quality of life; BSIqol: Barretos Short Instrument for Assessment of Quality of Life; EORTC QLQ-C30: European Organization for the Research and Treatment of Cancer Quality of Life Questionnaire Core 30; FACT-G: Functional Assessment of Cancer Therapy-General; ESAS: Edmonton Symptom Assessment System; BCH: Barretos Cancer Hospital; KMO: The Kaiser-Meyer-Olkin; ICC: Intraclass correlation coefficient; ECOG-PS: Eastern Cooperative Oncology Group performance status; TSDS: Total Symptom Distress Score.

## Competing interests

The authors declare that they have no competing interests.

## Authors' contributions

CEP and BSRP conceptualized the study and developed the questionnaire. CEP and FCR obtained the data. CEP analyzed the data. All authors provided input on the interpretation and they read and approved of the final draft of the manuscript.

## Supplementary Material

Additional file 1 **Figure S1.** The Barretos short instrument for assesment of quality of life (BSIqol) in Portuguese – original instrument. Click here for file

Additional file 2 **Figure S2.** The Barretos short instrument for assesment of quality of life (BSIqol) in English – translated instrument. Click here for file

Additional file 3 **Table S1.** Patient characteristics. Click here for file

Additional file 4 **Table S2.** Spearman correlation analysis between BSIqol domains and Performance Status and financial income. Click here for file
