# Supplementary figures and images for: The Barretos short instrument for assessment of quality of life (BSIqol): development and preliminary validation in a cohort of cancer patients undergoing antineoplastic treatment
Source: Health Qual Life Outcomes. 2012 Nov 29;10:144. doi: 10.1186/1477-7525-10-144 (PMC3541104; doi:10.1186/1477-7525-10-144)

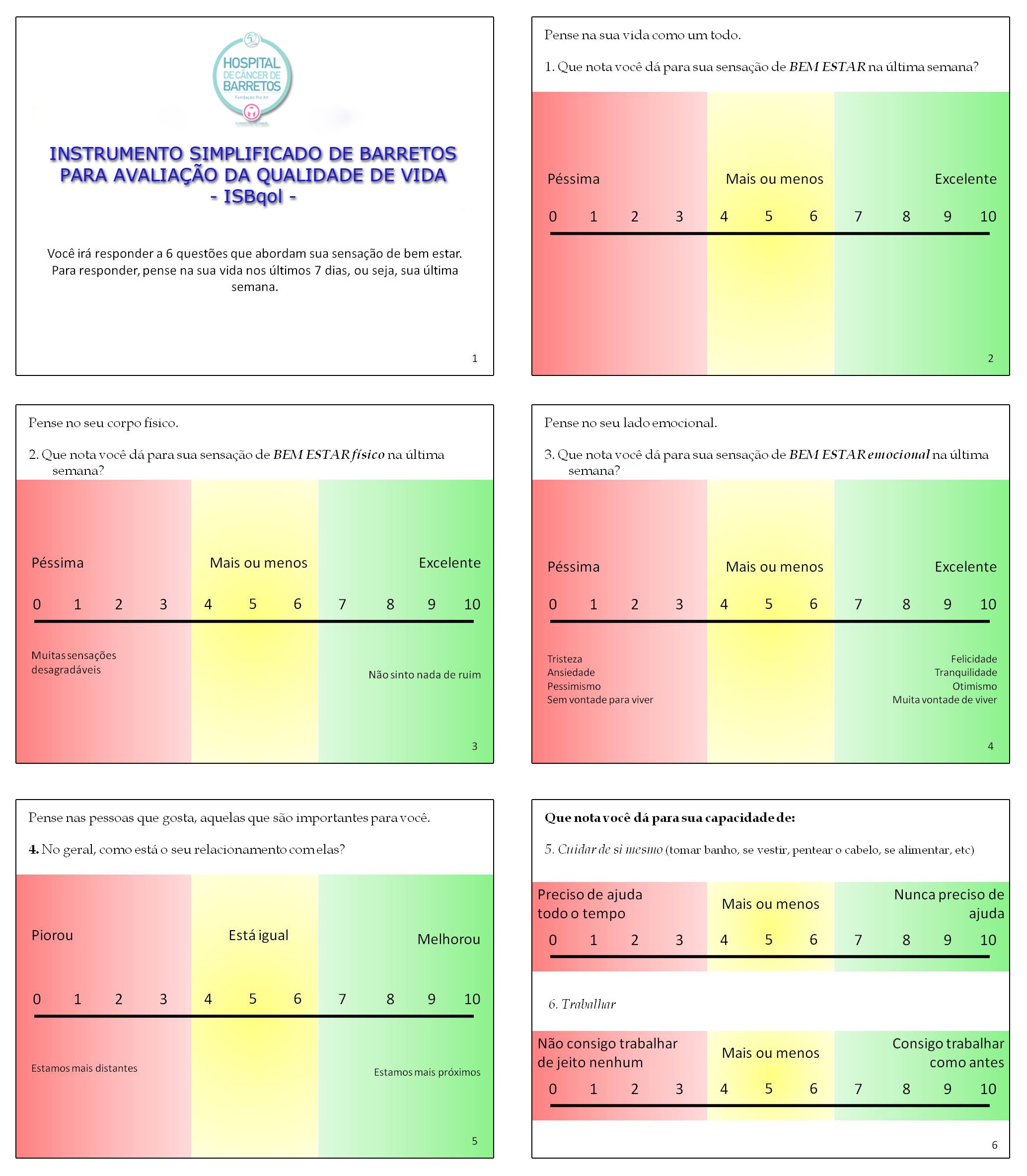

Supplement: Additional file 1 — Figure S1. The Barretos short instrument for assesment of quality of life (BSIqol) in Portuguese – original instrument. [file 1477-7525-10-144-S1.jpeg]
