# Supplementary material for: The Barretos short instrument for assessment of quality of life (BSIqol): development and preliminary validation in a cohort of cancer patients undergoing antineoplastic treatment
Source: Health Qual Life Outcomes. 2012 Nov 29;10:144. doi: 10.1186/1477-7525-10-144 (PMC3541104; doi:10.1186/1477-7525-10-144)
Supplement: Additional file 2 — Figure S2. The Barretos short instrument for assesment of quality of life (BSIqol) in English – translated instrument. [file 1477-7525-10-144-S2.pdf]

The Barretos Short Instrument for assessment of quality of life
# - BSIqol -

You will answer 6 questions regarding your sense of well-being.
In order to respond, think about the last 7 days of your life, that is, your last week

Think about your life as a whole.

1. What grade would you give to your sense of ***WELL-BEING*** during the last week?

Terrible | More or less | Excellent

0 1 2 3 4 5 6 7 8 9 10

Think about your physical body.

2. What grade would you give to your sense of *physical* ***WELL-BEING*** during the last week?

Terrible

More or less

Excellent

0 1 2 3 4 5 6 7 8 9 10

Several unpleasant feelings

I don't feel anything bad

Think about your emotional side.

3. What grade would you give to your sense of *emotional* ***WELL-BEING*** during the last week?

Terrible

More or less

Excellent

0 1 2 3 4 5 6 7 8 9 10

Sadness
Anxiety
Pessimism
No will to live

Happiness
Easiness
Optimism
Strong will to live

Think about the people you love, those that really matter to you.

4. Overal, how is your relationship with them?

Has gotten worse | The same | Has gotten better

0 1 2 3 4 5 6 7 8 9 10

We are more distant from one another

We are closer to one another

What grade would you give to your ability to:

5. *Take care of yourself* (bathing, dressing up, combing your hair, eating, etc)

I need help all the time				More or less				I never need help		
0	1	2	3	4	5	6	7	8	9	10

6. *Work*

I cannot manage to work at all				More or less				I am able to work as I did before		
0	1	2	3	4	5	6	7	8	9	10
