# Supplementary material for: The Barretos short instrument for assessment of quality of life (BSIqol): development and preliminary validation in a cohort of cancer patients undergoing antineoplastic treatment
Source: Health Qual Life Outcomes. 2012 Nov 29;10:144. doi: 10.1186/1477-7525-10-144 (PMC3541104; doi:10.1186/1477-7525-10-144)
Supplement: Additional file 3 — Table S1. Patient characteristics. [file 1477-7525-10-144-S3.docx]

**Supplementary Table 1.** Patient characteristics.

| **Variable** | **Patients** | |
| --- | --- | --- |
|  | **No** | **%** |
| *Age (years)* |  |  |
| Mean (SD) | 57.3 (11.7) |  |
| Median (range) | 58 (23-83) |  |
| *Family income ^a^* |  |  |
| Mean (SD) | 2.5 (3.8) |  |
| Median (range) | 2 (1-25) |  |
| *Gender* |  |  |
| Male | 23 | 29 |
| Female | 57 | 71 |
| *Marital status* |  |  |
| Married | 45 | 56 |
| Not married ^b^ | 35 | 44 |
| *Race/ethnicity* |  |  |
| White | 65 | 81 |
| Black | 10 | 13 |
| Asiatic | 1 | 1 |
| Non classifiable | 4 | 5 |
| *Work activity* |  |  |
| Active | 25 | 31 |
| Inactive | 54 | 68 |
| Unknown | 1 | 1 |
| *ECOG-PS* |  |  |
| 0 | 34 | 43 |
| 1 | 35 | 44 |
| 2 | 9 | 11 |
| 3 | 2 | 3 |
| *TNM Stage* |  |  |
| I | 13 | 16 |
| II | 14 | 18 |
| III | 15 | 19 |
| IV | 38 | 48 |
| *Educational level* |  |  |
| Illiterate | 8 | 10 |
| At least elementary education | 42 | 53 |
| At least high school | 19 | 24 |
| College or post graduation | 10 | 13 |
| Unknown | 1 | 1 |
| *Tumor site* |  |  |
| Breast | 38 | 48 |
| LGI | 20 | 25 |
| Prostate | 6 | 8 |
| UGI | 5 | 6 |
| Cervix | 2 | 3 |
| Ovarian | 2 | 3 |
| Skin and soft tissue | 2 | 3 |
| Unknown primary | 2 | 3 |
| H&N | 1 | 1 |
| Bladder | 1 | 1 |
| Uterine | 1 | 1 |
| *Type of treatment* |  |  |
| Chemotherapy | 40 | 50 |
| Hormone therapy | 40 | 50 |

Legend: ^a^ Expressed as one minimum wage (Brazil) = approximately US $344.9 on 07 June 2012. ^b^ including widowed and divorced. ^c^ Illiterate or not completed elementary education. ^d^ At least completed elementary education.
