# Supplementary material for: The Barretos short instrument for assessment of quality of life (BSIqol): development and preliminary validation in a cohort of cancer patients undergoing antineoplastic treatment
Source: Health Qual Life Outcomes. 2012 Nov 29;10:144. doi: 10.1186/1477-7525-10-144 (PMC3541104; doi:10.1186/1477-7525-10-144)
Supplement: Additional file 4 — Table S2. Spearman correlation analysis between BSIqol domains and Performance Status and financial income. [file 1477-7525-10-144-S4.docx]

**Supplementary Table 2.** Spearman correlation analysis between BSIqol domains and Performance Status and financial income

| **EORTC QLQ-C30** | **BSIqol** | | | | |
| --- | --- | --- | --- | --- | --- |
|  | **Global** | **Physical** | **Emotional** | **Functional** | **Social** |
| ECOG-PS | -0.650** | -0.533** | -0.433** | -0.714** | -0.224* |
| Financial income ^a^ | 0.065 | -0.001 | 0.037 | 0.048 | 0.208* |

*p<0.05 **p<0.01. **_**Underline indicates scores that should correlate theoretically.

Legend: ECOG –PS= Eastern Cooperative Oncology Group performance status.

^a^ Expressed as one minimum wage (Brazil) = approximately US $344.9 on 07 June 2012.
